# Dual statistical models link baseline visual attention measure to risk for significant symptomatic concussion in sports

**DOI:** 10.2217/cnc-2023-0002

**Published:** 2024-01-16

**Authors:** Lisa A Spielman, Jun Maruta, Jamshid Ghajar

**Affiliations:** 1 Department of Rehabilitation & Human Performance, Icahn School of Medicine at Mount Sinai, New York, NY 10029, USA; 2 Department of Neurology, Icahn School of Medicine at Mount Sinai, New York, NY 10029, USA; 3 Brain Trauma Foundation, Palo Alto, CA 94301, USA

**Keywords:** mild traumatic brain injury, oculomotor, predictive modelling, preseason assessment, sports concussion

## Abstract

**Aim:**

Athletic pre-season testing can establish functional baseline for comparison following concussion. Whether impacts of future concussions may be foretold by such testing is little known.

**Materials & methods:**

Two sets of models for a significant burden of concussion were generated: a traditional approach using a series of logistic regressions, and a penalized regression approach using elastic net.

**Results:**

3091 youth and adult athletes were baseline-assessed. 90 subsequently experienced concussion and 35 were still experiencing a significant burden of concussion when tested within two weeks. Both models associated prior history of head injury and visual attention-related metrics with a significant burden of concussion.

**Conclusion:**

Pre-season testing of visual attention may identify athletes who are at risk for significant sports-related concussion.

## Plain language summary

Athletic pre-season testing can establish functional baseline for comparison following concussion and may predict impacts of future concussions. In this study, 3,091 youth and adult athletes were baseline-assessed. 90 subsequently experienced concussion and 35 were still experiencing a significant burden of concussion when tested within two weeks. A statistical model and a machine-learning model both associated prior history of head injury and visual attention-related metrics with a significant burden of concussion. Pre-season testing of visual attention may identify athletes who are at risk for significant sports-related concussion.

## Tweetable abstract

Pre-season testing of visual attention may identify athletes who are at risk for significant sports-related concussion

Concussion is a common occurrence in high school and collegiate sports, with overall rates reported between 0.23 and 0.43 per 1000 athletic exposure respectively [1]. Various premorbid predictors of sports-related concussion and prolonged recovery have been studied [2–6], but the findings related to factors that may increase the risk and burden of concussion are sparse and inconsistent [7–10]. Recent attempts to model risk have shown modest utility and relatively poor effect sizes [11]. Most individuals recover from concussion within weeks [12–14], but even a brief period of symptoms may interfere with return to play, work, school, and everyday routines and obligations. Identifying athletes at risk for significant post-concussion symptoms allows for prognostic counseling and may guide return to play via targeted training approaches.

Here, we retrospectively analyzed data from a larger concussion-related project with a particular focus on a normative characterization of attention-dependent oculomotor performance in a variety of military and civilian groups using a broad assessment battery [15,16]. The project was motivated by the premise that attention dysfunction is a common sequela of a concussion [17–19]. Some of the results from this project, not overlapping with the current study, have been published elsewhere [20–23]. In the current study we address the possibility that a deficit in attentional reserve makes one more prone to a significant, high symptom report, concussion.

Attention is multi-faceted and supported by interdependent neural subprocesses [24,25]. It is also known that there is a large overlap between the neural networks for attentional and oculomotor controls [26–28]. Bringing and maintaining an object of interest on the fovea with saccade and smooth pursuit eye movements are not an automatic response to retinal inputs but are guided by complex processes including attention, memory and expectation [27–29]. This account is closely associated with the profound dependence of human vision on capturing selective images on the fovea and the required precision in eye movement control to achieve this goal. In sports, critical to performance is sustained attention via anticipatory neural coordination, i.e., the ability to dynamically synchronize visual input and motor output [30,31]. Pursuit eye movement demands dynamic synchronization of external visual events and eye movement commands by forming internal spatiotemporal prediction of the target trajectory [30,32]. Since eye movements can be easily, accurately, and objectively recorded, eye movement performance metrics of predictive pursuit may provide an objective assessment of anticipatory attention function [30].

The extensive battery of baseline assessments allowed us to consider a broad variety of domains as potentially influencing the burden of a concussion. In addition to oculomotor performance, we assessed neurocognitive functioning, psychological and mood symptoms. We measured the presence of symptoms common to concussion in all cases, whether or not there was a prior reported head injury, to determine their global predictive power. Importantly, we avoided the reliance on restrictive inclusion criteria that is common in similar investigations, instead retaining subjects with history of head injury, with attention-deficit/hyperactivity disorder (ADHD), and with several other conditions that might otherwise preclude entry into research, strengthening the generalizability of our findings. Although ADHD specifically is recognized as a risk factor for concussion and a possible predictor of poorer outcomes [33–37], in this study, we regard the condition as part of more generalizable attention issues rather than an isolated confounder.

The large pool of potential explanatory variables available in a baseline battery such as ours can pose analytic challenges to identify those with predictive utility. Standard linear and logistic regression techniques may suffer from problems of overfitting and multicollinearity, even when using stepwise selection [38]. Penalized regression approaches such as least absolute shrinkage and selection operator (LASSO) and elastic net address these problems by setting a limiting factor that constrains the sum of the regression coefficients, reducing the number of retained variables by setting regression coefficients to zero for unnecessary variables. The method is impervious to multicollinearity; further, elastic net improves on LASSO by allowing more precise determination of the limiting factor [39].

While penalized regression techniques are not new, they are somewhat underutilized in the literature on clinical and applied medicine. An additional objective of this study is to demonstrate the differences in model output between the standard and penalized regression approaches. We will present the approaches in parallel and then compare the fitted models directly. We expected that the penalized regression approach would show both procedural (i.e., more straightforward variable selection, reduced data manipulation) and explanatory (i.e., superior fit and predictive indices) advantages compared with the standard approach.

The aims of the current study were (a) to identify which factors assessed at baseline, if any, were associated with increased acute symptom burden of concussion during the subsequent season, and (b) to explore the model outputs of a machine-learning approach compared with more traditional analytic methods. These aims were examined by building as broad a model as possible based on an extensive pre-season dataset from a highly generalizable group of athletes, some of whom subsequently experienced concussive injury.

## Materials & methods

### Study population

Male and female athletes were recruited via coordinated outreach effort to schools and community organizations. The inclusion criteria were participation in organized competitive athletic activity, age 12–30 years, normal or corrected-to-normal vision, and for athletes over the age of 18 years, a high school diploma or equivalent, or expected timely high school graduation. Potential participants were screened for eligibility through telephone and in-person interviews. All participants were required to have good English proficiency. Individuals were excluded for eye-sight abnormalities precluding assessment, such as amblyopia, uncorrected near- or far-sightedness and macular degeneration.

### Study protocol

The subject enrollment, consent process, and testing protocols were approved by the institutional review boards of Weill Cornell Medical College (NY, USA) and Stanford University (CA, USA). In collaboration with school, university and community athletic organizations in respective local areas, athletes were enrolled and baseline tested. Prior to data collection, written informed consent by adult subjects, or legal guardians of minor subjects with the minors' assent, was obtained in accordance with the Declaration of Helsinki. Participants subsequently identified by a trainer, coach, or other team staff with concussion underwent post-injury testing within 2 weeks of injury. A concussion was defined as an event of blunt impact on the head, with loss of consciousness, post-traumatic amnesia, or at least one of the following symptoms: dizziness, nausea, headaches, balance problems, blurred or double vision, or feeling dazed/confused. Although for the purpose of this research we did not rely on formal medical diagnosis of concussion necessary for clinical management of the injury, this definition is consistent with the guidance of the American Academy of Neurology [40]. Data collection spanned from September of 2012 through September of 2016.

### Primary measures

#### Neurocognitive testing

The Simple Reaction Time (SRT) module from the Automated Neuropsychological Assessment Metrics Version 4 (ANAM) [41] and the Trail Making test (TRAILS) [42]. The SRT module was specifically chosen for its previously reported potential utility in concussion management [43,44]; other ANAM modules were not deployed.

#### Symptom questionnaires

The Rivermead Post-concussion Symptoms Questionnaire (RPQ) [21,45] and the Brain Injury Screening Questionnaire (BISQ) [46].

Part II of the BISQ contains a list of 100 self-reported common post-concussion symptoms representing four factors: attention-memory, mood/anxiety/depression, impulsivity/aggression, and physical symptoms. Average scores across the items in each factor ranged from 0 to 3 determined in terms of the probability of exposure to traumatic brain injury (TBI), with 0 indicating no probability (i.e., no symptoms) and 3 high probability (i.e., consistent symptoms). Norms were established by the instrument's authors based on the ability to distinguish known cases of TBI from depression, chronic pain and normal control cases; a cutoff of 0.5 SD above the TBI mean from the normative data was identified as an appropriate cutoff to distinguish TBI. For this study, we focused on the attention-memory score which according to the instrument's authors is the score that best differentiates TBI from other groups [46,47], and is based on mean frequency of experiencing 28 common post-concussion symptoms such as ‘thinking slowly’, ‘difficulty making decisions’ and ‘reading slowly’. The attention-memory factor score cutoff of 0.53, the score corresponding to the cutpoint recommended by the study authors, was used to identify athletes with moderate-severe post-concussive symptom burdens at the time of testing [47].

#### Psychological & mood scales

Center for Epidemiological Studies Depression Scale (CES-D) [48]; Beck Anxiety Inventory (BAI) [49]; BAI-Youth (BAI-Y) [50]; Beck Depression Inventory-Youth (BDI-Y) [50]; Conners Adult ADHD Rating Scales Self-report: Short Version (CAARS) [51]; and Conners 3rd Edition (Conners 3) for pediatric ADHD [52] (Pearson Clinical, San Antonio, TX).

#### Predictive pursuit eye movement

Pursuit eye movement performance was assessed with a circular tracking paradigm, following the protocol described previously [30]. Data were collected binocularly at a 500 Hz sampling rate using a video-based eye tracker (EyeLink 1000, SR Research Ltd., Mississauga, Canada) under normal room lighting. The stimulus was a target moving along a circular trajectory with a 10° radius at 0.4 Hz on a computer monitor. Collected eye movement data were screened with an automated algorithm for >10% of missing data, artifacts associated with inadequate quality of calibration, or poor head stabilization during recording as evidenced by a large change in visual fixation records and were also visually inspected [15,20]. Performance was characterized in terms of the instability of the gaze on the target (standard deviations [SDs] of gaze positional errors in radial and tangential directions of the target trajectory), average radial error, average phase lag or lead relative to the target, and the ratios of smooth pursuit velocity to the target velocity in the horizontal and vertical directions (horizontal and vertical gains).

### Statistical analysis

We conducted two analytic approaches in parallel: a traditional method using a series of logistic regression models, and a machine learning approach using penalized regression. While machine learning analyses are slowly gaining popularity in medical research, there remain concerns regarding the purely data-driven nature of the method, as opposed to the more theory- or hypothesis-guided nature of traditional approaches. We aimed to examine whether the two approaches would demonstrate converging findings, and to evaluate the differences among the methods. Throughout, our focus is on comparing the model outputs produced by the two analytic approaches, rather than an in-depth review of the statistical properties of each (but see [38,39]).

We took a sequential approach to narrow down the large set of baseline measures for use in a streamlined final model. The pre-injury characteristics were grouped into sets of construct-consistent domains: demographic characteristics, head injury history, circular pursuit eye movement parameters, cognitive assessments, common concussion symptoms, and psychological and mood symptoms. In a series of multiple logistic regression models, each set of candidate variables was examined for the ability to distinguish between athletes with significant post-concussion symptoms and those without concussion. Individual variables within each domain were retained for testing in the final summary model if they met the threshold p < 0.10 defined in terms of the null hypothesis that the candidate variable had no relation to the symptomatic versus non-concussion grouping. We used this threshold for retention on the basis that, for the purposes of model specification, the risk of type II error was of higher concern than type I error, since candidate variables that did not act as useful predictors of injury would be discarded after examination of the summary model. Odds ratios (ORs) were calculated to determine effect sizes. We constructed a summary model based on the variables retained from each of the domain-specific models. The final set of variables in this summary model was examined for its ability to predict, based on baseline assessments, which athletes would experience post-concussive symptoms following subsequent head injury.

This sequential approach was used to address a common problem: narrowing down a large set of variables – many of which were intercorrelated – to a number appropriate to the size of the sample, while avoiding the problem of multicollinearity. Recent adaptations of machine learning techniques suggested an alternate approach to identifying which of the large set of baseline characteristics might show utility as a predictor of concussion. Penalized regression approaches such as LASSO are used to identify a tuning parameter, lambda, that applies shrinkage to the estimated coefficients such that some approach 0 and are dropped from the model. Elastic net is a related technique that has been shown to outperform LASSO with highly correlated predictors [39]. Given the strong association among concussion-related symptoms in our predictor set, we chose this latter approach. We randomly split the dataset into 70% training data and 30% testing data, and then entered the full set of baseline predictors into a logistic elastic net regression model with tenfold cross validation on the training dataset. After identifying the optimal lambda to determine the final set of predictors, we applied the resulting model to the testing dataset. We then tested the elastic net-determined set of predictors in a standard logistic regression to directly compare the findings to the traditional approach described above, examining pseudo R^2^ and misclassification errors as indices of predictive utility. Finally, we submitted both logistic regression logits (traditional and elastic net-derived) to area under the curve (AUC) analysis on their receiver operating characteristic curves to determine the relative explanatory power of the final fitted models [53,54]. Analyses were performed in SPSS v.25 (IBM SPSS Statistics for Windows, Version 25.0., NY, USA) and Stata 16 (Stata Statistical Software: Release 16, TX, USA).

## Results

A total of 3091 athletes underwent baseline testing and 90 (2.9%) were reported to have sustained a concussion. This rate was roughly comparable to those reported in the literature [1,55,56]. The post-concussion assessment for one athlete occurred outside of the 14-day window; this participant was excluded from analysis. As reported previously [21], the remaining 89 athletes were evaluated within 2 weeks following injury, with testing ranging from the day of injury to 14 days after injury ($\bar{\text{x}}$ = 5.8 ± 3.5).

**Figure 1.**
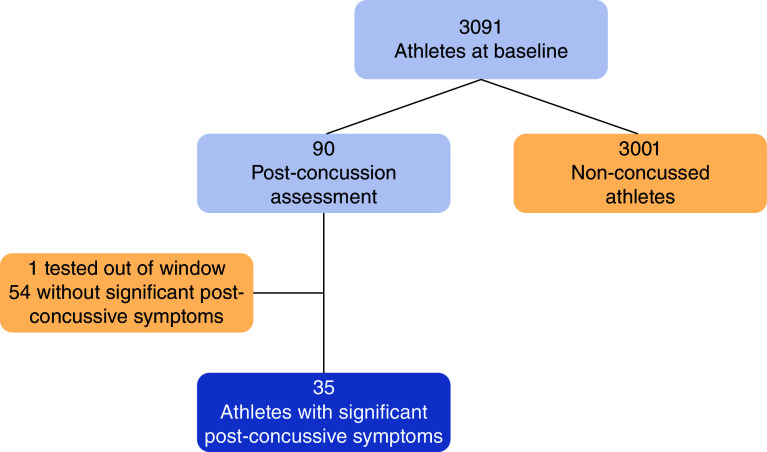
Participant flow diagram.

It was expected that time since injury would contribute to recovery. However, this expectation was not confirmed. Using the BISQ Attention-Memory subscale, 35 cases (39% of concussion cases, 1.1% of the total sample) were identified as experiencing post-concussive symptoms at the time of testing, and the remaining 54 cases were identified as having their initial symptoms resolved before the testing. If athletes had been impacted by their concussion in a random and unbiased fashion and recovering gradually, those identified as still experiencing significant symptoms at the time of testing should have been more likely to be among the ones tested early within the 14-day window, yet there was no difference in time-to-testing between the two groups (t[87] = 0.47, p = 0.64). The BISQ subscale scores plotted against the time between injury and testing had a slope of 0.0002 per day, which was non-significantly different from zero (t[87] = 0.012, p = 0.99). Thus, to study the impacts of concussions in athletes, we chose the final sample consisting of 35 concussed athletes and 3001 non-concussed athletes for a total n = 3036 (Figure 1).

### Characteristics of the sample

Athletes were likely to be Caucasian (53%), and male (62%). Age ranged from 12–30 years ($\bar{\text{x}}$ = 18.6 ± 3.0), with 11% aged 12–14 years, 16% aged 15–17 years, and the remaining 73% aged 18 years and over. About a quarter (28%) reported a prior head injury, 9% reported having a learning disability, 7% reported psychiatric diagnosis, and 4% reported a neurological diagnosis. Non-concussed athletes and athletes with significant post-concussive symptoms did not differ on any of these characteristics, but the latter group of athletes were younger (p = 0.032) and had a higher rate of prior head injury (about half with post-concussive symptoms vs about one quarter of non-concussed; p = 0.002) (Table 1). Data on the timing of prior head injury were not collected.

**Table 1.** Sample characteristics.

	Non-concussed athletes	Athletes with significant post-concussive symptoms
Age (years), $\bar{\text{x}}$ (SD)	18.6 (3.0)^†^	17.5 (2.9)^†^
History of head injury	27.5%^†^	51.4%^†^
Caucasian	52.9%	40.0%
Male	62.1%	51.4%
Learning disability	8.5%	8.6%
Psychiatric disorders	7.2%	8.6%
Neurological disorders	3.5%	5.7%

† p < 0.05.

### Preliminary models

The following five logistic regression models were examined for candidate variables' ability to distinguish between athletes with significant post-concussion symptoms from those without concussion.

#### Model 1: demographic & head injury history

Candidate variables were: age; history of head injury; race; sex; and learning disability. Age (p = 0.06, OR = .874) and history of head injury (p = 0.001, OR = 3.47) were retained.

#### Model 2: circular pursuit eye movement parameters

Note eye movement records of 821 cases were deemed invalid by the automated screening algorithm, and were dropped from this analysis. We retained these cases in the other preliminary models since we had not yet determined if any of the circular pursuit parameters would be included in the final model. Individuals missing eye movement data did not differ significantly on any study variables except that missing cases were younger (p < 0.001) and had a lower rate of prior head injury (p = .005), which reinforces that missingness was likely not due to cognitive factors. 817 of the 821 were non-concussed. Candidate variables were: SD radial; SD tangential; mean radial error; mean phase error; horizontal gain; and vertical gain. SD radial (p = 0.04, OR = 4.59) was retained.

#### Model 3: cognitive assessments

Candidate variables were: SRT median reaction time (RT); SRT SD of RT; SRT throughput; TRAILS A time; TRAILS B time; TRAILS A errors; TRAILS B errors; TRAILS A standard score; and TRAILS B standard score. None met the threshold for retention.

#### Model 4: symptoms common to concussion

Candidate variables were individual items from the RPQ: headaches; dizziness; nausea; noise; sleep; fatigue; irritable; depressed; frustrated; forgetfulness; concentration; longer to think; vision; light; double vision; and restlessness. We also considered the three additional items included in our implementation of RPQ (Maruta *et al.* 2018a): balance; mental fogginess; and drowsiness. Noise (p = 0.036, OR = 1.63), sleep (p = 0.078, OR = 1.39) and irritable (p = 0.049, OR = 1.59) were retained.

#### Model 5: psychological & mood symptoms

Candidate variables were: BAI anxiety; BAI-Y anxiety; CES-D depression; BDI-Y depression; Conners 3 inattention; Conners 3 hyperactive/impulsivity; Conners 3 learning problems; Conners 3 defiance/aggression; Conners 3 family relations; CAARS inattention; CAARS hyperactivity/restlessness; CAARS impulsivity; CAARS problems with self-concept; and CAARS ADHD Index. BAI anxiety (p = 0.05, OR = 1.08) and Conners 3 inattention (p = 0.05, OR = 1.04) were retained.

### Summary model

Taking preliminary models into account, a summary model was created to combine all candidate predictors that met the p < .10 criteria. Measures that differed between adult and pediatric cases were harmonized based on Z scores (for BAI/BAI-Y) or T scores (for Connors 3/CAARS) for inclusion into the summary model. The final model is presented in Table 2. The overall model was significantly associated with the likelihood of significantly symptomatic concussion (*χ*^2^ [8] = 40.53, p < 0.001, pseudo R^2^ = 0.13), with several individual baseline characteristics showing significant associations with taxing post-concussive symptoms.

**Table 2.** Summary model of selected candidate predictors.

	B	SE	p-value	Odds ratio	95% CI
Age	-0.13	0.07	0.055	0.875	0.76–1.00
History of head injury	1.15	0.38	0.003	3.152	1.49–6.66
SD radial	0.94	0.39	0.016^†^	2.553	1.19–5.46
ADHD inattention^‡^	0.04	0.02	0.043^†^	1.035	1.00–1.07
Anxiety^§^	-0.06	0.17	0.728	0.944	0.68–1.31
Noise^§^	0.39	0.20	0.047^†^	1.479	1.00–2.18
Sleep^§^	0.20	0.19	0.292	1.217	0.85–1.75
Irritable^§^	0.11	0.20	0.565	1.120	0.76–1.65

† p < 0.05.

‡ Standardized subscale score from Connors/CAARS.

§ RPQ items.

CI: Confidence interval; SE: Standard error.

Prior head injury (OR = 3.15, p = 0.003) and increased SD radial, representing gaze instability (OR = 2.55, p = 0.016) showed the strongest associations with the presence of significant post-concussive symptoms following injury. Baseline SD radial scores did not differ for those with (0.668 ± 0.323) or without (0.679 ± 0.291) prior head injury (t(2251) <1, ns), so the predictive utility of SD radial does not appear to be related to incomplete recovery from prior injury. Further, baseline SD radial scores were unrelated to baseline sleep complaints (r = -0.002, ns), ruling out sleep deprivation as an explanatory factor. ADHD Inattention (OR = 1.04, p = 0.043) and noise sensitivity (OR = 1.48, p = 0.047) also were also significantly associated with semi-persistent symptoms following concussion. Age is marginally significant (OR = 0.88, p = 0.055), suggesting that younger age is associated with taxing post-concussive symptoms. Preinjury anxiety, sleep symptoms, and irritability did not significantly increase the likelihood of experiencing post-concussive symptoms, but their presence increased the overall predictive ability of the model and so they were retained.

### Elastic net analysis

We ran the elastic net analysis on the 70% training dataset, which contained 1551 cases after missing data were omitted. Two criteria determined selection of the optimal lambda and associated model coefficients: minimizing the Bayesian information criterion (BIC) and maximizing variance explained, operationalized as the in-sample deviance ratio. After evaluating these criteria, we selected a model with alpha = 0.50, lambda = .0027, BIC = 279.55, deviance ratio = 0.108. Retained predictors for the model is presented in Table 3.

**Table 3.** Elastic net model on training sample.

	Coef.	Odds ratio
History of head injury	0.58	1.79
SD radial	0.32	1.37
ADHD inattention^†^	0.16	1.17
Age	-0.14	0.87
Noise^‡^	0.19	1.21
Light^‡^	-0.07	0.93
Dizziness^‡^	0.11	1.12
TMT A Errors	-0.01	0.99
TMT B Errors	-0.24	0.78

† Standardized subscale score from Connors/CAARS.

‡ Rivermead items.

CI: Confidence interval; SE: Standard error.

The results are strikingly similar to the summary model using standard logistic regression: history of head injury, ADHD inattention, SD radial, age, and noise are again retained as predictors, along with two additional common concussion symptoms, dizziness and light sensitivity, as well Trails A and B Errors. Applying the model to the 30% testing dataset of 662 cases, the model retained consistent explanatory power, with deviance ratio = 0.11.

Standard logistic regression results of the final model for the full sample, using the predictors derived from the elastic net model, are presented in Table 4. The overall model was significant (*χ*^2^ [9] = 48.35, p < .001, pseudo R^2^ = 0.15). History of head injury, ADHD inattention, SD radial, and trails B Errors were statistically significant predictors of a concussion burden, with age and noise marginally significant (p = 0.057 and 0.056, respectively).

**Table 4.** Logistic regression from elastic net model on full sample.

	B	SE	p-value	Odds ratio	95% CI
History of head injury	1.18	0.38	0.002^†^	3.27	1.54–6.95
SD radial	0.89	0.38	0.020^†^	2.43	1.15–5.14
ADHD inattention^‡^	0.04	0.02	0.012^†^	1.04	1.01–1.08
Age	-0.13	0.07	0.057	0.88	0.77–1.00
Noise^§^	0.45	0.24	0.056	1.57	0.99–2.49
Light^§^	0.15	0.23	0.519	1.16	0.74–1.83
Dizziness^§^	0.09	0.24	0.713	1.09	0.69–1.73
TMT A errors	-0.39	0.41	0.337	0.67	0.30–1.51
TMT B errors	-0.65	0.30	0.027^†^	0.52	0.29–0.93

† p < 0.05.

‡ Standardized subscale score from Connors/CAARS.

§ RPQ items.

CI: Confidence interval; SE: Standard error.

### Comparison of models

Given the overlap in retained variables, predictive capability of the two models are quite close, with the elastic net model performing slightly better: pseudo R^2^_summary_ = 0.13 and pseudo R^2^_elastic net_ = 0.16. We calculated AUC for the summary and elastic net logits. Again the elastic net model was slightly superior: AUC_summary_ = 0.76 and AUC_elastic net_ = 0.78. Notably, the elastic net approach required many fewer steps to arrive at a comparable model, skipping the cumbersome iterative method used to narrow down sets of predictors in the traditional approach.

## Discussion

We examined a large and broadly generalizable cohort of athletes to provide evidence for pre-existing and measurable baseline characteristics associated with risk of taxing symptoms if concussed during the ensuing season. The potential predictive characteristics represented a diverse set of factors including demographics; current and prior physical, neurological, psychiatric, and mood conditions; standardized neurocognitive testing; and, unique to this investigation, pursuit eye movement. Inclusion of the pursuit eye movement task allowed us to examine the particular role of anticipatory attention in the burden of concussion. The specific pursuit eye movement metric identified in the models, SD radial, has been previously associated with sleep deprivation and cognitive fatigue [57,58]; the current findings suggest its utility in predicting an increased burden of sports-related concussion.

We compared results built on traditional and machine-learning-based analytic techniques. The approaches led to models of similar content and explanatory power, reinforcing confidence in the findings. The elastic net regularized regression model is an illustration that these techniques have great utility in the clinical and applied medicine literature, and we hope its use here will inspire more widespread adoption of the technique. These findings are novel in the ability to identify premorbid factors that are specifically associated with an increased symptom burden during the acute post-concussion phase, as opposed to examining any occurrence of head injury whether or not it results in semi-persistent symptoms [59].

To be clear, the models presented here do not provide a causal explanation for sports-related concussion. Rather, the models highlight the key role of attention in an early phase of post-concussion trajectory. Most self-reported symptoms resolve quickly, but this outlook does not mean that concussion is resolved [60]. We thus selected 35 athletes who were still experiencing a significant burden of concussion at the time of testing so that we would be able to isolate predictive baseline characteristics. Indeed, the only statistically significant predictors common to both final models besides prior head injury were two attention-related variables, namely, SD radial and ADHD Inattention. It is possible that a prior head injury is related, perhaps bi-directionally, to attention problems in that the deficit in attentional reserve at baseline could potentially be the result of a prior head injury. We were not able to delve into such relations because of the limitation in the sample size, but effects of repeat concussion remain an important question. The design of the study, which assessed over 3,000 athletes, led us to expect a larger pool of athletes with significant post-concussion symptoms. What is good news clinically can be challenging from a research perspective. While most of the concussed athletes were expected to make a good functional recovery, presently we were not able to study longitudinal outcomes. Still, we believe that the current findings point to fruitful areas for further investigation.

Other study limitations include the specialized sample – this study examined concussion in young athletes and it is unclear whether these findings are generalizable to varieties of mechanisms of injury or other ages. Eye movement was recorded binocularly at a high sampling rate using research-grade equipment, but technological advancements are easing access to such measures. At the time of data collection, we lacked the means to screen for the validity of eye movement records. High-resolution eye tracking technology is evolving. Improvements in eye tracking technology supplemented by immediately screening the data with an automated algorithm should reduce the technical failure rate. Further research is needed to validate these findings in heterogeneous populations in a variety of settings including non-athletes. Additionally, further studies are needed to determine whether training strategies addressing deficits in eye movement subsequently reduce subsequent head injury and symptom burdens.

## Conclusion

A better understanding of different post-concussion trajectories should improve individual athletes' readiness to deal with the period of transition to recovery or chronic symptom development. This study is novel in asking whether a preseason assessment can improve the prediction of post-concussion trajectories. Comprehensive testing including visual attention at the beginning of season may identify athletes who are at risk for an increased symptom burden of sports-related concussion. Understanding baseline attention characteristics may aid prognostic counseling.

**Summary points**

- We examined a large and broadly generalizable cohort of athletes to provide evidence for pre-existing and measurable baseline characteristics associated with risk of significant symptoms if concussed during the ensuing season.
- We compared results built on traditional and machine-learning based analytic techniques.
- Both traditional and machine-learning modeling approaches associated prior history of head injury and visual and other attention-related metrics with a risk of significant concussion symptoms.
- Findings are novel in the ability to identify premorbid factors that are specifically associated with a risk of increased symptom burden during the acute post-concussion phase.
- Objective visual attention testing at the beginning of season may identify athletes are at risk for an increased burden of sports-related concussion.
- Understanding the baseline characteristics in athletes is key to direct prognostic counseling.
- Machine-learning based analytic techniques have great utility in clinical and applied medicine.
